# Beauty is context-dependent: Naturalness, familiarity, and semantic meaning influence the appreciation of geometric shapes

**DOI:** 10.1177/20416695241303004

**Published:** 2024-12-26

**Authors:** Georgiana Juravle, Charles Spence

**Affiliations:** Sensorimotor Dynamics Laboratory, Faculty of Psychology and Education Sciences, 112930 Alexandru Ioan Cuza University, Iasi, Romania; Crossmodal Research Laboratory, Department of Experimental Psychology, Oxford University, Oxford, UK

**Keywords:** ice, symmetry, Platonic solids, sensory, temperature, crossmodal correspondences

## Abstract

Rounded shapes are associated with softness and warmth, whereas Platonic solids are associated with hardness and coldness. We investigated the temperature-shape association through sensorial/conceptual qualities of geometric ice-like textured shapes. In Experiment 1, participants viewed symmetrical rotating 3D shapes (five Platonic solids—cube, tetrahedron, octahedron, icosahedron, dodecahedron; a star polyhedron and a sphere) and control shapes (naturalistic and angular), rating them in terms of liking, hardness, temperature, wetness, and texture. In Experiment 2, participants visualized ice, and selected/rated, from 22 adjectives, those corresponding to the concept of ice. In Experiment 3, for each of the shapes from Experiment 1, participants chose the most appropriate conceptual attribute from among the six attributes most frequently reported in Experiment 2. All shapes looked cold. Liking and hardness ratings were similar for the ice-sphere and the Platonic solids, with an enhanced liking and the attribution of the “beautiful” concept for starlike ice shapes. The cube was appreciated as solid and the Platonic solids as strong and bright. Self-reported introversion, extroversion, and fitness level were significantly related to the appreciation of geometric ice structures. These findings are discussed in relation to crossmodal correspondences and the role of individual differences.

An extensive body of evidence links geometric shapes to various sensorial and conceptual qualities. We most often visually judge the various objects that we come into contact with based on their geometric properties, such as shape and curvature, but also their orientation, size, and volume (Kahrimanovic et al., 2010). When manipulating and/or haptically interacting with objects of interest, other important material object information is also extracted/becomes available, including the object's weight, temperature, and texture, with these characteristics often derived through direct contact with the skin (Gallace & Spence, 2014). Other senses, such as audition, make significant, if rather unexpected contributions to material perception (e.g., as outlined in the multisensory experience of judging an object's material qualities, termed “Shitsukan” in Japanese; see Fujisaki, 2020; Komatsu & Goda, 2018; Spence, 2020a; see also Guest et al., 2002; Spence & Zampini, 2006, for further audio-tactile interactions). Through repeated exposure, objects of interest also become associated with higher-order conceptual attributes, such as connotative meaning linked to objects we regularly interact with (Osgood, 1952; Osgood et al., 1957; see also Gombrich, 1960, 1972; Marks, 2014, on the crossmodal meaning of symbols).

With respect to easily recognizable shapes, there exist some specific manufactured structures, e.g., the ancient stone-carved spheroids, that have successfully spanned hundreds of thousands of years starting from the Middle Paleolithic, with scientists still puzzled by these spheroids' intended function/purpose (Muller et al., 2023; see also MacGregor, 2000). Nowadays, rounded shapes have received an extensive consideration in the literature, with many findings consistently demonstrating people's preference for objects presented in rounded, over angular forms (Bar & Neta, 2006; Ghoshal et al., 2015; Silvia & Barona, 2009). One explanation for this liking response is in terms of *symmetry*: The perfectly symmetrical sphere is significantly preferred when compared to other symmetrical geometric shapes (e.g., see Turoman et al., 2018). Recently, the sphere was contrasted with other symmetrical objects that have been considered beautiful (namely the Platonic solids, as described by Plato in his dialogue *Timaeus*), and people's liking was probed, together with other types of sensorial appreciation for geometric shapes, such as their perceived hardness, and their expected temperature. Note that for Plato, beauty was intrinsic to the geometric regularity and symmetry of these solids, and he used them creatively so as to represent the elements of the material world, in order to construct/propose a theoretical model of the universe. There are five Platonic solids (tetrahedron, octahedron, icosahedron, cube/hexahedron, and dodecahedron), rendered as regular convex polyhedra with all faces formed of regular congruent polygons meeting at each vertex. The key characteristic of the Platonic solids is that they are all *perfectly symmetrical*, irrespective of their direction of rotation. The sphere is preferred, being evaluated as the softest and receiving the warmest temperature ratings, with the Platonic solids, by contrast, rated as harder and significantly colder than the sphere (Juravle et al., 2022). While earlier conceptual pairings of the *words* circle and square have been congruently related to *concepts* of warmth/coldness, as well as softness/hardness (Liu & Kennedy, 1993, 1997), this round *shape*—warm *temperature* correspondence highlighted in Juravle et al.’s (2022) study, is, to the best of our knowledge, the first empirical demonstration for the shape-temperature crossmodal correspondence. To validate this crossmodal correspondence, studies were designed in our lab that manipulated the humanly perceptually available (estimated) temperatures (i.e., temperatures we are comfortably interacting with) for geometric shapes. For example, *taste*-wise, hot-temperature round pieces of food were recently shown to be preferred to rectangular ones (Juravle et al., 2024). With the present study, our goal is to further investigate the shape-temperature crossmodal correspondence for cold estimated temperatures. In this respect, we concentrate on the geometric shape of objects, as their material properties are kept constant and they are rendered visually to appear as if made of ice. The approach outlined here targets the *naturalness* of geometric shapes, our *familiarity* with them, and the *semantic meaning* that they carry.

With Plato's categorization of the so-called Platonic solids as the most beautiful shapes (Lloyd, 2010; see also Melo, 2022; Wilczek, 2015), as well as Plato's attribution of specific natural worldly elements to each of them (e.g., fire for the tetrahedron, air for the octahedron, water for the icosahedron, and earth for the cube; the dodecahedron was conceived to represent the whole universe), the question arises as to the specificity and *naturalness* of the typical testing of liking and other sensorial qualities in geometric shape perception. Note that most studies to date have assessed shape perception using traditional 2D renderings on a computer screen (though see Etzi et al., 2012, for a study looking at manipulating 3D shapes), with the further specifics of texture and/or object materiality in the context of its practical purpose for daily activities receiving little, if no attention. In this respect, the physical characteristics of objects such as their shape symmetry and (perceived) shape naturalness may be closely interconnected to their material perception. Take, for a recent example, the mathematically derived geometric infinite polyhedra (Huylebrouck, 2023; see also Bertol, 2016, for further visual conceptualizations of the Platonic solids), which, devoid of specific material properties, appear, if anything, to be less beautiful than the Platonic solids that served as their basis. To uncover the beauty of the Platonic solids, given their naturalness (i.e., as designated by their presence in the defining geometric shapes that exist in nature, both living forms, e.g., in the DNA of the simplest life forms, and nonliving forms, e.g., the structure of crystals), one needs to assess them with respect to their *natural materiality*. Furthermore, by taking into consideration previous findings of the appreciation of Platonic solids as appearing visually as colder in temperature (Juravle et al., 2022), the present study investigates naturalness in geometric shape perception by assessing these geometric shapes of interest in an ice-textured form, i.e., a naturally appearing material that everyone is familiar with.^1^ In Experiment 1, because of their material natural ice-like presentation, it was hypothesized that the Platonic solids would be liked significantly more than other geometric shapes (no matter whether they were symmetrical or asymmetrical). Specifically, we aimed to further explore Platonic perfect symmetry. For this, we used a naturalistic asymmetrical stone-like shape as a control (see Juravle et al., 2022) and also introduced two shapes outside the theorized Platonic perfect symmetry: the symmetrical regular star, together with the angular asymmetrical star-like control shape used as its control. Because the control geometric shapes that we presented are not universally recognized and/or proposed as being beautiful^2^ (e.g., as is the case for the sphere and the Platonic solids), the expectation was of higher esthetic appreciation for the sphere and the Platonic solids, as well as a lower estimated temperature and enhanced hardness for the Platonic solids (Juravle et al., 2022).

It is not only an object's material characteristics that influence and drive our appreciation for its beauty. Beauty is often in the eye of the beholder, and as such, individual subjective characteristics need to be taken into account. In this respect, *familiarity* with certain geometric shapes may be expected to influence liking and sensorial assessments. For example, people show an enhanced preference for objects with curved contours they are familiar with, with this appreciation modulated by individual traits, such as affective intuition and unconventionality (Chuquichambi et al., 2021). Moreover, other individual differences, such as autistic traits, have recently been described in shape-based crossmodal correspondences: Specifically, when given the choice, people with high autistic traits are less likely to choose consensual crossmodal color-shape associations, such as the pairing of colors red/pink with rounded shapes, or the pairing of the sweet taste with color pink (Chen et al., 2021; see Spence, 2022a, for a review). Therefore, in the experiments reported here, we formulated exploratory hypotheses with regard to the specific personality traits targeted in the three studies, with the starting point being the ice texture. The exploratory hypothesis that was put forward with respect to ice consumption in everyday life, in the form of drinks “on the rocks,” targeted introversion/extroversion personality traits because of the typical social environment in which such drinks are consumed. Relatedly, because of people's preference for cold drinks (Eccles et al., 2013; cf., Hyde & Witherly, 1993), the exploration of a link toward sensation seeking in personality could be considered (Spence, 2022b). Furthermore, we also assessed fitness by self-report because of the strength component of fitness that we expected to mirror the texture/consistency of the Platonic *solids*. Note that all these were formulated as exploratory hypotheses, to have an additional measure of any latent underlying individual differences that could help explain and potentially underlie the appreciation of geometric shapes.

## Experiment 1

### Methods

#### Participants

An a priori power calculation with an α = .05 and 1 − β* *= .80 indicated that a sample size of 36 participants would be sufficient to detect an *f *= 0.25 effect in the data (G*Power 3.1; Erdfelder et al., 2009; Faul et al., 2007), should one be present (Cohen, 1988). A total of 38 participants (27 females, mean age of 31 years, *SD* = 9 years, age range 19–52 years) were therefore recruited to take part in Experiment 1. The participants were recruited from socializing apps (e.g., WhatsApp, Facebook) and gave their informed consent to take part in the study. The study was approved by the Ethics Board of the Alexandru Ioan Cuza University of Iasi, Romania (no. 2325/24.05.2021). This study conforms to the Declaration of Helsinki and to all subsequent amendments (Declaration of Helsinki, 1964, 2013).

#### Materials and Apparatus

**Figure 1.**
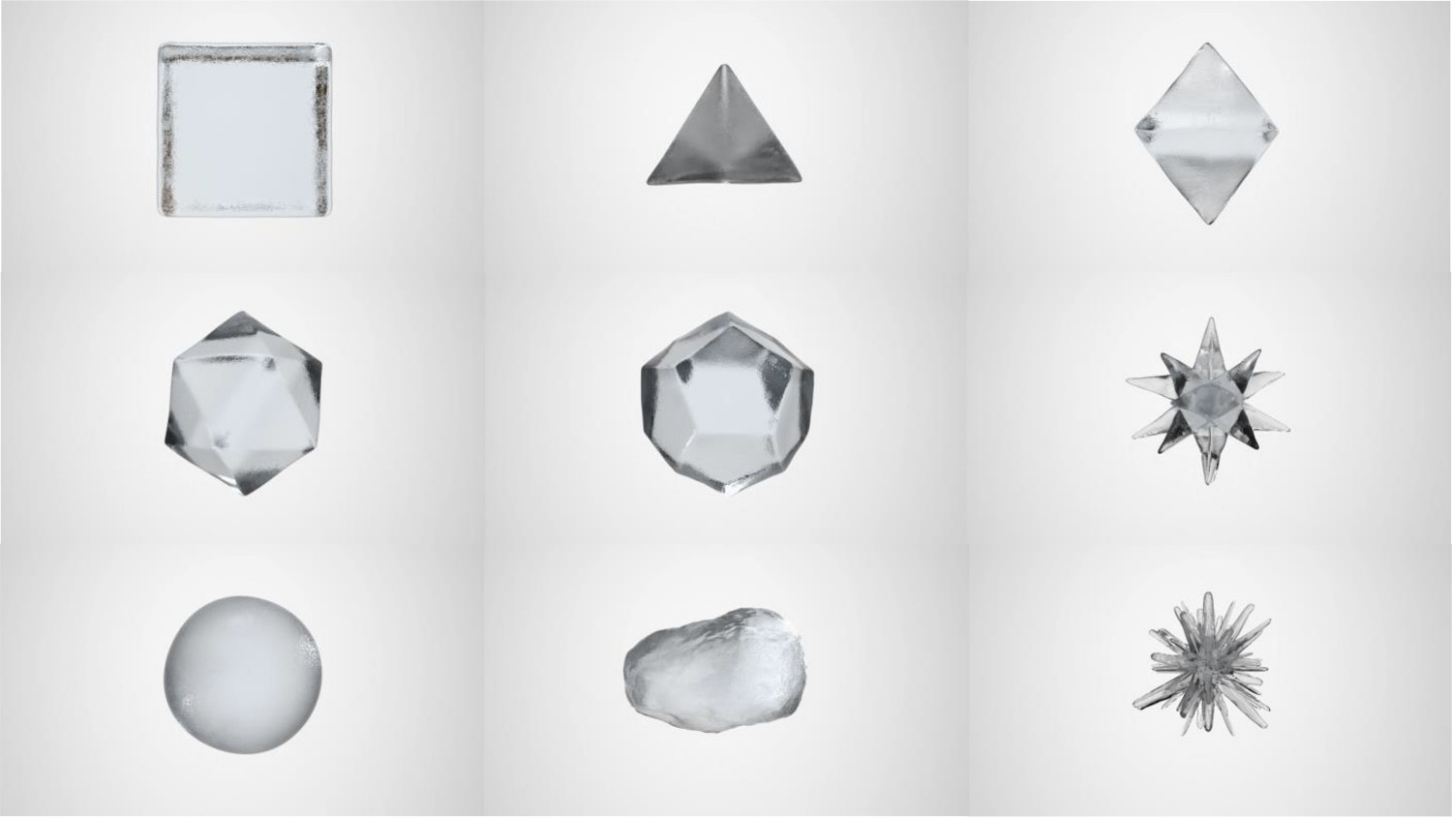
Still pictorial examples of the experimental stimuli used in Experiment 1. From the upper left corner: cube, tetrahedron, octahedron (upper row), icosahedron, dodecahedron, star polyhedron (middle row), sphere, naturalistic irregular control shape, and angular irregular shape (lower row). Note that participants assessed the stimuli while they were in rotational motion (see Supplemental Material for the actual rotating versions of the visual stimuli).

The study was conducted online on the Testable platform (www.testable.org; Rezlescu et al., 2020) between 21 May 2023 and 10 July 2023. The experimental stimuli consisted of nine GIF images representing the different geometric shapes: the five Platonic solids (a cube, a tetrahedron, an octahedron, an icosahedron, and a dodecahedron), a regular star polyhedron, a sphere, one naturalistic irregular shape modeled on a natural rock, and another irregular angular shape. All visual stimuli were created for the specific purpose of the study. The stimuli were modeled with the help of 3ds Max 2023 (e.g., the Noise modifier and the Soft select tools, Autodesk, San Francisco, USA) and rendered in V-Ray (Version 6, Chaos, Sofia, Bulgaria). All of the geometric shapes were derived from a cube with a side of 100 cm and a camera view positioned 370 cm in front. The geometric shapes had the same ice appearance and the same height (6.7 cm/6°23′ degrees of visual angle, assuming a standard viewing distance of 60 cm from monitor). The objects were designed to have a hard, wet, and transparent-like-ice *visual appearance*, for an objective (visual) perceptual experience of ice. The complete image (object + background) presented to the participants on each trial in Testable full-screen mode was 20.5 cm high × 12 cm wide. All of the geometric shapes rotated clockwise continuously in the GIF file and throughout the experimental trial, the 3D geometric shape was perceived in continuous rotating motion (see Figure 1 for static 2D examples of the geometric shapes used in Experiment 1). The experimental stimuli, together with the data that support the findings of this study, are all available from the corresponding author (G.J.), upon request. The actual rotating stimuli used in Experiment 1 are presented in the Supplemental Material.

#### Procedure and Experimental Design

At the beginning of the experiment, the participants were asked to enter into full-screen mode and calibrate their screen, such that all participants saw the experimental stimuli at the same size. The experimental platform presented the participants with instructions outlining the general scope of the study and the type of data that would be collected as a result of their taking part. They were informed that they could quit at any point, gave their informed consent, and provided the required demographic data. Each experimental trial consisted of the presentation of a single experimental stimulus (i.e., a rotating geometric shape) together with a visual analog scale (VAS), positioned below the rotating geometric figure. The participants used their mouse to move the pointer on the scale and rate the presented shape on the given VAS, with only the end anchors visible. The VASs used were set from 0 to 100 points, with 0 = *lack of the measured variable*, 50 = *moderate presence*, and 100 = *maximum recorded*.

The manipulated independent variable was geometric shape (cube, tetrahedron, octahedron, icosahedron, dodecahedron, star polyhedron, sphere, naturalistic irregular shape, and angular irregular shape). Each geometric shape was presented until a response was detected, for each of the five VASs recorded as dependent measures: estimated *Liking* (*very unpleasant* to *very pleasant*), estimated *Hardness* (*very soft* to *very hard*), estimated *Temperature* (*very cold* to *very hot*), estimated *Wetness* (*very dry* to *very wet*), and estimated *Texture* (*not at all slippery* to *very slippery*). In a repeated measures design, three repetitions were recorded for each visual stimulus. In total, each participant completed 135 trials in a randomized order.

Following the experimental trials, the participants had to fill in a series of psychological scales. Specifically, from the International Personality Item Pool (Goldberg et al., 2006; Iliescu et al., 2015), the scale assessing *extroversion* was used, with 10 items including “I am the life of the party,” “I feel comfortable around people,” “I start conversations,” “I talk to a lot of different people at parties,” “I don't mind being the centre of attention,” “I don't talk a lot,” “I keep in the background, I have little to say,” “I don't like to draw attention to myself,” and “I am quiet around strangers.” Participants responded on a 5-point Likert scale with anchors 1 (*completely disagree*) and 5 (*completely agree*). In the calculation of the final extroversion score, the last five items were reversed; the total extroversion score is the sum of the 10 items used. The total extroversion score had excellent internal consistency (Cronbach's α = .91; 95% CI [0.86, 0.95]).

From the Big Five personality traits (McCrae & John, 1992), *introversion* was measured on a four-item subscale including the items “I am reserved,” “I am sometimes shy, inhibited,” “I am talkative,” and “I am outgoing, sociable.” To provide a measure of introversion, the last two items were reversed (e.g., see Tuovinen et al., 2020). Participants responded on a 5-point Likert scale with the anchors 1 (*completely disagree*) and 5 (*completely agree*). The total introversion score was calculated as the sum of the four items; Cronbach's α = .86 (95% CI [0.75, 0.92]) was obtained, which is considered good internal consistency.

The participants also received a five-item questionnaire that assessed their self-reported level of *physical fitness*, the International Fitness Scale (IFIS; see Ortega et al., 2011, 2023). The scale includes items assessing overall self-repoted physical fitness, cardiorespiratory fitness, muscular force, speed/agility, and flexibility, on a 5-point scale from 1 (*poor*) to 5 (*very good*). The calculated internal consistency for this study is excellent (Cronbach's α = .92; 95% CI [0.87, 0.95]). The experiment lasted for about 20 min.

#### Data Analysis

The data from each of the five VASs (Liking, Hardness, Temperature, Wetness, and Texture) were inspected visually with histograms and sample/theoretical quantiles (Q-Q) plots, together with skewness and kurtosis calculations. In order to compare the Platonic solids to the sphere and the control shapes, all of the VASs were analyzed with one-way repeated measures analysis of variance (ANOVAs) with the factor of geometric shape (cube, tetrahedron, octahedron, icosahedron, dodecahedron, star polyhedron, sphere, the naturalistic irregular shape, and angular irregular shape). These ANOVAs were followed up by Holm-corrected paired-samples *t*-tests to identify any geometric shape effect in the data (Holm, 1979). The Greenhouse-Geiser correction was used to adjust the ANOVA degrees of freedom, when the sphericity assumption was violated.

Separate correlations were performed between the average data for the five sensorial scales collected in Experiment 1 and the total scores calculated for introversion and extroversion, as well as participants' overall self-reported fitness. Statistical analyses were performed in Matlab (R2021a, MathWorks, Natick, MA, USA) and JASP 0.16.3 (JASP Team, 2022).

### Results

#### Liking

**Figure 2.**
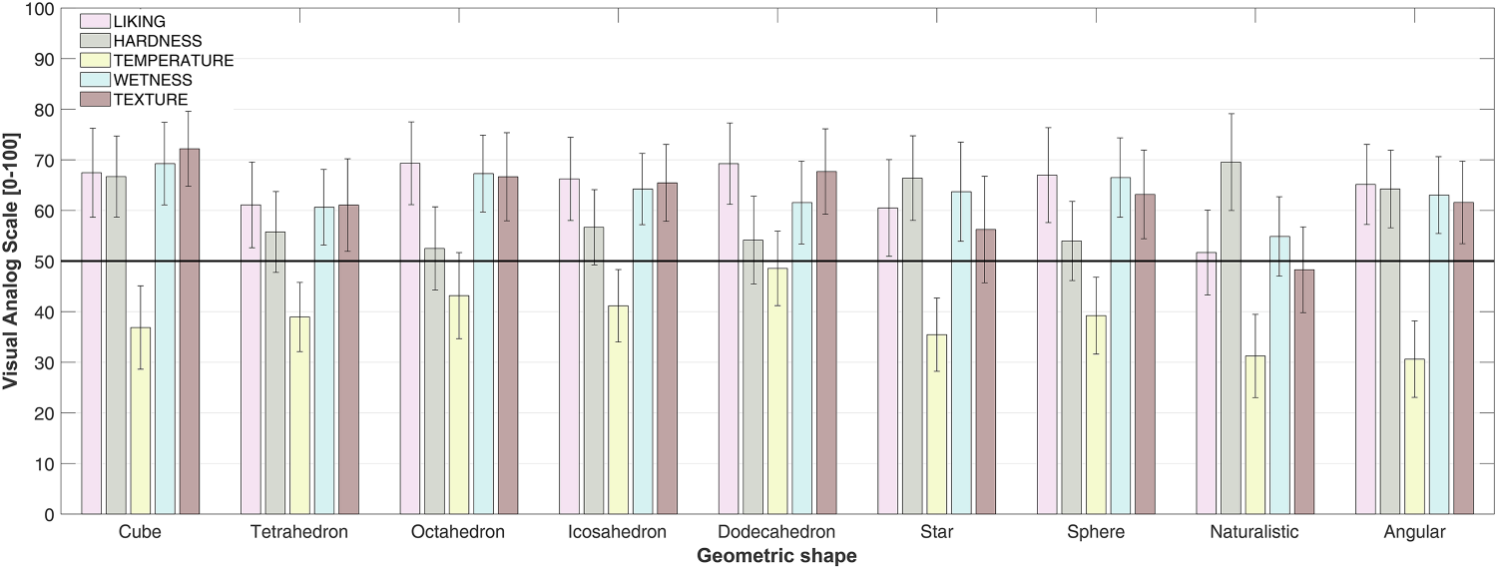
Means ± 95% CI for each of the five VASs used to evaluate the geometric shapes in Experiment 1: Liking (0 = *very unpleasant* to 100 = *very pleasant*), Hardness (0 = *very soft* to 100 = *very hard*), Temperature (0 = *very cold* to 100 = *very hot*), Wetness (0 = *very dry* to 100 = *very wet*), and Texture (0 = *not slippery at all* to 100 = *very slippery*). The thick horizontal line indicates the midpoint of the VAS scale [refer to the online version of this graph for color version].

The results indicated a significant main effect of geometric shape, *F*(4.36, 161.24) = 3.13; *p *= .002; $\eta_{p}^{2}=0.078$ ; *ε *= .545, with the naturalistic shape (*M *= 51.68; *SE *= 3.91) being significantly less liked than the cube, *M *= 67.46; *SE *= 4.34; *t*(37) = 3.50, *p *= .019; *d *= 0.605, the octahedron, *M *= 69.32; *SE *= 4.06; *t*(37) = 3.91, *p *= .004; *d *= 0.676, the icosahedron, *M *= 66.24; *SE *= 3.97; *t*(37) = 3.23, *p *= .045; *d *= 0.558, and the sphere, *M *= 66.98; *SE *= 4.14; *t*(37) = 3.39, *p *= .026; *d *= 0.587. None of the other post hoc *t*-tests reached significance (see Figure 2).

#### Hardness

The results highlighted a significant main effect of geometric shape, *F*(3.92, 144.93) = 3.66; *p *= .008; $\eta_{p}^{2}=0.090$ ; *ε *= .490, indicating that the naturalistic shape (*M *= 69.55; *SE *= 4.72) was rated as looking significantly harder than the octahedron, *M *= 52.49; *SE *= 4.06; *t*(37) = −3.48, *p *= .021; *d *= −0.683, and was also rated as marginally significantly harder when compared to the sphere, *M *= 53.97; *SE *= 4.72; *t*(37) = −3.18, *p *= .057; *d *= −0.624; see Figure 2. Importantly, the sphere did not differ significantly in terms of its expected hardness from any of the Platonic solids, nor from the regular star polyhedron and the angular irregular shape (*p *= n.s.).

#### Temperature

The results of Experiment 1 indicated a significant main effect of geometric shape on temperature evaluations, *F*(4.86, 179.76) = 4.03; *p *= .002; $\eta_{p}^{2}=0.098$ ; *ε *= .607; see Figure 2. Post hoc tests highlighted that the dodecahedron (*M *= 48.55; *SE *= 3.63) was rated as significantly warmer than the naturalistic shape, *M *= 31.25; *SE *= 3.73; *t*(37) = 4.33, *p *< .001; *d *= 0.743, and the angular irregular shape, *M *= 30.61; *SE *= 3.57; *t*(37) = 4.49, *p *< .001; *d *= 0.770. A trend toward the octahedron (*M *= 43.17; *SE *= 4.20) being rated as warmer than the irregular shape was also apparent in the temperature data, *t*(37) = 3.14, *p *= .061; *d *= 0.539.

#### Wetness

The results highlighted a significant main effect of geometric shape, *F*(3.97, 146.71) = 2.27; *p *= .023; $\eta_{p}^{2}=0.058$ ; *ε *= .496, indicating that the naturalistic shape (*M *= 54.87; *SE *= 3.75) was rated as looking significantly dryer than the cube, *M *= 69.25; *SE *= 4.063; *t*(37) = −3.62, *p *= .013; *d *= 0.593, and marginally significant dryer when compared to the octahedron, *M *= 67.28; *SE *= 3.75; *t*(37) = 3.12, *p *= .070; *d *= 0.512; see Figure 2.

#### Texture

The results highlighted a significant main effect of geometric shape, *F*(3.09,114.31) = 3.64; *p *= .014; $\eta_{p}^{2}=0.089$ ; *ε *= .386, indicating that the naturalistic shape (*M *= 48.28; *SE *= 4.32) was rated as looking significantly less slippery than the cube, *M *= 72.18; *SE *= 3.66; *t*(37) = 4.61, *p *< .001; *d *= 0.911, the octahedron, *M *= 66.66; *SE *= 4.30; *t*(37) = 3.54, *p *= .016; *d *= 0.701, the icosahedron, *M *= 65.45; *SE *= 3.75; *t*(37) = 3.31, *p *= .035; *d *= 0.654, and the dodecahedron, *M *= 67.68; *SE *= 4.17; *t*(37) = 3.74, *p *= .008; *d *= 0.740. A marginally significant difference in texture was also registered between the cube and the star polyhedron, *M *= 56.24; *SE *= 4.32; *t*(37) = 3.07, *p *= .074; *d *= 0.608; see Figure 2.

Lastly, means together with standard deviations for the three scales of extroversion, introversion, and self-reported physical fitness are presented in Table 1.

**Table 1.** Means with standard deviations recorded in Experiments 1–3 for the three questionnaires assessing introversion, extroversion, and overall self-reported fitness level.

	Experiment 1		Experiment 2		Experiment 3	
	Mean	*SD*	Mean	*SD*	Mean	*SD*
Introversion	10.53	3.80	9.07	3.18	11.49	3.88
Extroversion (IPIP)	30.53	7.91	38.20	8.30	32.08	8.97
Fitness (IFIS)	20.95	4.90	17.49	4.38	15.13	4.10

*Note.* Introversion from the Big Five personality traits. IPIP = International Personality Item Pool; IFIS = International Fitness Scale.

**Figure 3.**
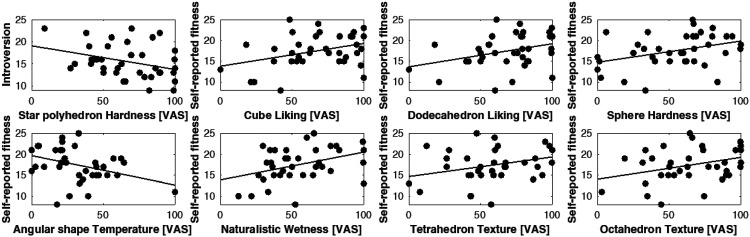
Scatter plots of significant correlations found in Experiment 1 between the self-reported introversion, self-reported fitness, and the sensorial qualities of geometric shapes.

The results indicated that extroversion was not related to any of the sensorial appreciation scales measured in Experiment 1 (all *p*s = n.s.). A significant correlation was, however, found between the participants' introversion (total score) and the reported hardness of the star polyhedron (*r *= −.337; *p *= .039), in that the higher the introversion score, the softer the star polyhedron was rated by the participants. Importantly, the self-reported fitness level significantly correlated with the liking of the cube (*r *= .368; *p *= .023) and the dodecahedron (*r *= .340; *p *= .037): The fitter the participants considered themselves to be, the likelier they were to positively appreciate these two regular shapes. Intriguingly, the higher the participants' self-reported fitness level, the harder they evaluated the consistency of the sphere (*r *= .373; *p *= .021). Though not significant, a similar marginally significant effect on hardness was also registered for the star polyhedron (*r *= .313; *p *= .056). Furthermore, our results indicate that the fitter the participants declared themselves to be, the more slippery they rated the tetrahedron (*r *= .326; *p *= .046) and the octahedron (*r *= .346; *p *= .033). Concerning the irregular shapes tested in Experiment 1, the results show that the fitter the participants (by self-report), the wetter they perceived the naturalistic rock-shaped ice to be (*r *= .383; *p *= .017). Importantly, a negative correlation is detected for the evaluated temperature data, in that the fitter the participants consider themselves to be, the colder they evaluate the temperature of the angular irregular-shaped piece of ice (*r *= −.387; *p *= .016; see Figure 3).

### Discussion

In agreement with the crossmodal symmetry-based associations that have been reported previously (Juravle et al., 2022; Turoman et al., 2018), the results of Experiment 1 highlight a clear effect of symmetry, with the Platonic solids in the shape of the cube, the octahedron, and the icosahedron, as well as the sphere, all being significantly more liked, than the naturalistic rock-like shape. However, in contrast to previous results suggesting that the sphere is typically the preferred geometric shape, the dominance of the sphere in terms of liking dissipates when this is presented in an ice format. Such a result might be taken to indicate that rounded ice shapes may appear unfamiliar and/or somehow to lack naturalness.

Not only is the naturalistic irregular geometric shape liked less, but it is also rated as being significantly harder than the octahedron, and marginally harder than the sphere. The naturalistic shape is also significantly dryer, as compared to the cube, with such a result suggesting an intrinsic wetness in the perception of symmetrically shaped ice cubes. Furthermore, symmetrical geometric shapes such as the cube, octahedron, icosahedron, and the dodecahedron are perceived as significantly more slippery textured and thus aligned to the intended feel of ice, as compared to the naturalistic irregular control shape.

As expected, all temperature assessments in Experiment 1 were situated below the moderate demarcation of the scale used in our study. In this context, it is the round-shaped Platonic solid dodecahedron, and not the sphere, that was rated as the warmest, relative to the other irregular geometric shapes that appeared as though they were made of ice. Furthermore, with no difference in hardness, wetness, or texture, between the sphere and the Platonic solids, it would appear that *visual* material cues play a significant role when people sensorially assess various geometric shapes. Note that when presented in a neutral hue, the sphere is typically perceived as being softer and also thermally rated as moderately warm (Juravle et al., 2022). As unexpected as this result may appear, such an even distribution in terms of liking and appreciation for sensorial attributes for both the rounded and angular symmetrical shapes could signal a rather context-based appreciation of shape. That is, just as the connotative associations of color have been shown to be context-dependent, as captured by the “color-in-context” theory (e.g., Elliot & Maier, 2012). In terms of shape, we may take the primitives of angularity and roundedness as universal signifiers of liking. However, context and individual differences could further be fitted as higher-order categories when affectively and sensorially appreciating shape (see also Chuquichambi et al., 2022, for a discussion).

In Experiment 1, regular versus irregular geometric shapes presented visually to appear as if they were made of ice were assessed for liking, together with the sensorial qualities of expected hardness, temperature, wetness, and slipperiness in texture. Having established the correspondence between the ice shapes and their expected sensorial qualities, Experiment 2 was designed to investigate whether any conceptual language-based correspondence would exist in the appreciation of ice. For this, participants were asked to mentally imagine/visualize ice and to select those adjectives that corresponded to the general concept of ice.

## Experiment 2

### Methods

Methods are similar to those used in Experiment 1; only the differences are outlined below.

#### Participants

An a priori power calculation with an α = .05 and 1 − β* *= .80 indicated that a sample size of 29 participants would be necessary to detect a *r *= .5 effect in the data (G*Power 3.1; Erdfelder et al., 2009; Faul et al., 2007), should one be present (Cohen, 1988). A total of 41 participants (23 females, mean age of 30 years, *SD* = 11 years, age range 16–53 years) took part in Experiment 2.

#### Materials, Procedure, and Experimental Design

The study was conducted online on Google Forms between 18 May 2023 and 16 June 2023. The participants were presented with a succession of 22 trials that required a single answer on a 1 (*not at all*) to 10 (*very much*) Likert scale assessing their appreciation of ice as being one of 22 diverse conceptual attributes, including fresh, clear, pure, colored, bright, powerful/strong, crystalline, transparent, opaque, dense, viscous, diluted, shiny, structured, beautiful, solid, fine, fluid, clean, natural, liquid, and heavy. They were asked to visualize the ice as each of the presented attributes/concepts and use the Likert scale to assess their appreciation of ice as the currently presented attribute/concept from each of the 22 selected attributes enumerated above. Following the experimental trials, the participants were asked to fill in the same succession of psychological scales, as those measured in Experiment 1: the scale assessing extroversion from the International Personality Item Pool, the total *extroversion* score had good internal consistency (Cronbach's α = .89; 95% CI [0.83, 0.93]); the *introversion* scale with an acceptable Cronbach's α = .72 (95% CI [0.55, 0.84]); and the IFIS fitness with good Cronbach's α = .86 (95% CI [0.77, 0.92]). The experiment lasted for about 5–10 min.

#### Data Analysis

Total preference scores were calculated as the sum of individual ratings for each of the 22 attributes, together with frequency tables. The attributes were ordered by their total sum (i.e., preference score), and those with the highest scores were used in Experiment 3. Finally, correlations were conducted between the conceptual attribute preference scores and the total scores for introversion, extroversion, and self-reported fitness.

### Results

**Figure 4.**
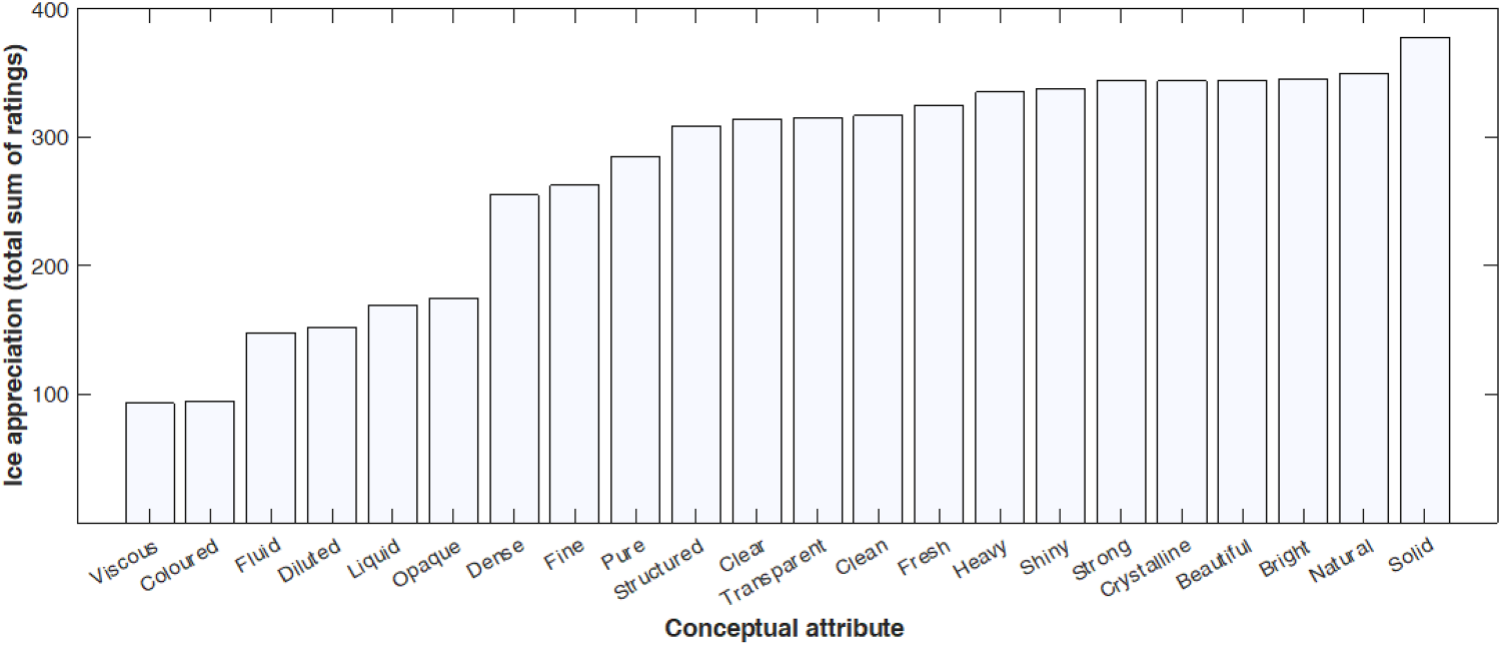
Histogram of the total sum of ratings for ice appreciation calculated across all participants in Experiment 2, for each of the 22 conceptual attributes that were presented.

Means together with standard deviations for the three scales of extroversion, introversion, and self-reported fitness are presented in Table 1. Figure 4 shows the distribution of total preference scores for the conceptual attributes tested in Experiment 2.

**Figure 5.**
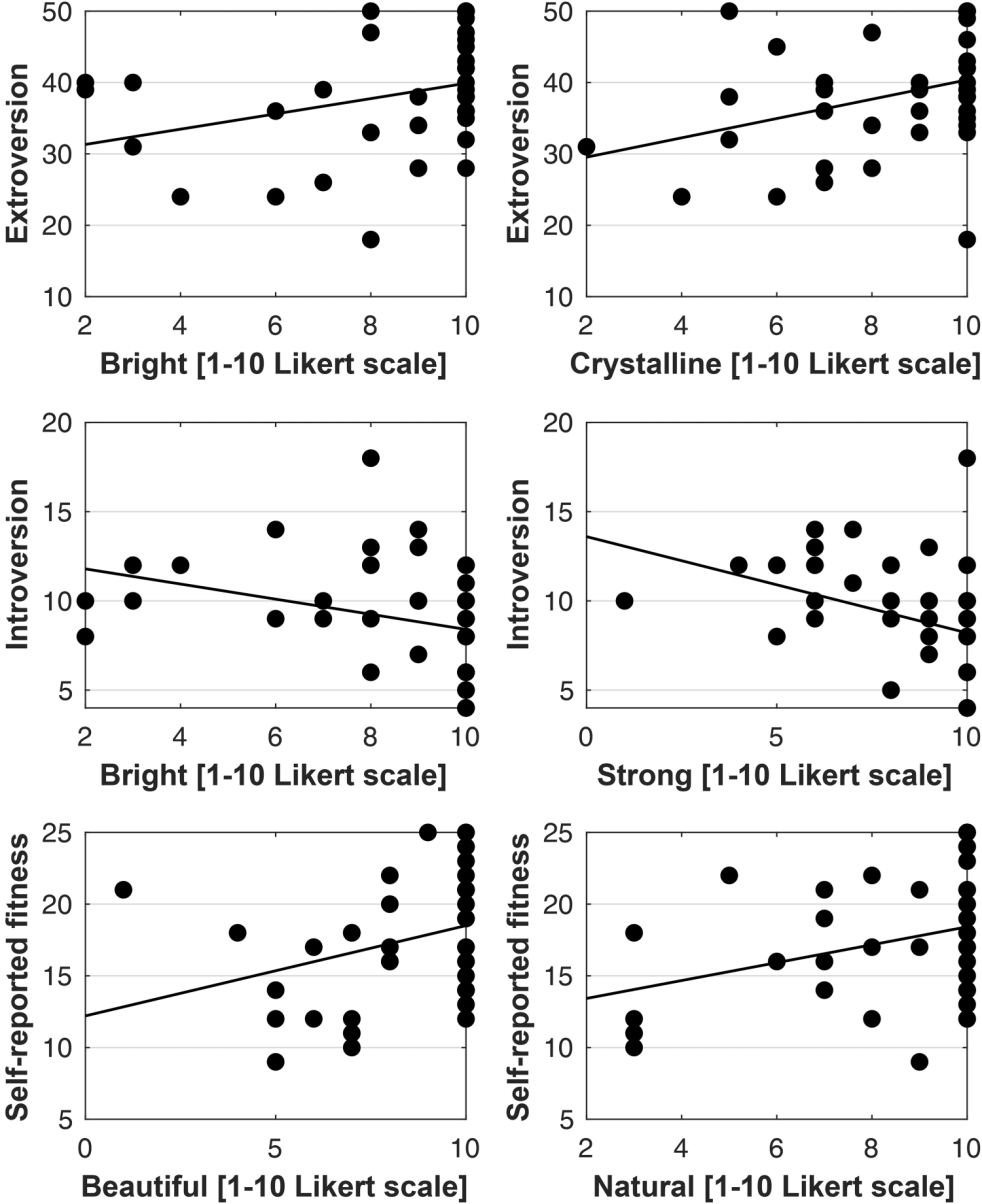
Scatter plots of significant correlations found in Experiment 2 between the self-reported extroversion, introversion, and self-reported fitness and the conceptual attributes of geometric shapes.

The results of Experiment 2 indicated that participants appreciated ice mostly as solid, natural, and bright, and with an equal rating: strong, crystalline, and beautiful. The more extroverted participants were, the higher their appreciation of ice as being bright (*r *= .312; *p *= .047) and crystalline (*r *= .336; *p *= .032). Relatedly, the less introverted participants were, the brighter (*r *= −323; *p *= .040) and stronger they rated ice as being (*r *= −.364; *p *= .019). Lastly, self-reported fitness positively correlated with participants' appreciation of ice as beautiful (*r *= .311; *p *= .048) and natural (*r *= .323; *p *= .039). See Figure 5 for a graphical representation of the significant correlations in Experiment 2.

### Discussion

The results of Experiment 2 provide an overview of the preferred qualities used when people conceptually evaluate ice. Other recent studies have established a conceptual link between various visually presented textures, e.g., furry versus crystalline visually presented textures, which were paired in associative learning tasks requiring classification of textures as either hot or cold in temperature, with words of fur/metal or happy/sad emojis (Barbosa Escobar, Wang, Corredor & Velasco, 2023a), highlighting an affective mechanism that is implicated in the appreciation of temperature (Barbosa Escobar, Velasco, Byrne & Wang, 2023b, 2023c).

To have an additional conceptual association for the ice shapes used in Experiment 1, we selected the most frequent conceptual attributes recorded in the appreciation of ice in Experiment 2. Specifically, in Experiment 3, the participants assessed each of the five Platonic solids, together with the star-shaped polyhedron, the sphere, and the control naturalistic and angular shapes used previously in Experiment 1, for the most frequent conceptual attributes found to be paired with ice in Experiment 2 (i.e., solid, natural, bright, strong, crystalline, and beautiful). Our expectation was for the identification of higher ratings of Platonic solids as being solid and strong, whereas, taking into consideration the known enhanced liking of rounded shapes (Bar & Neta, 2006; Ghoshal et al., 2015; Silvia & Barona, 2009), we expected the sphere to be evaluated as significantly more beautiful.

## Experiment 3

### Methods

An a priori power calculation with an α = .05 and 1 − β* *= .80 indicated that a sample size of 143 participants was necessary to detect an effect size of *w *= 0.30, in the data (G*Power 3.1; Erdfelder et al., 2009; Faul et al., 2007), given that an effect was present (Cohen, 1988). A total of 177 participants received course credit for taking part in Experiment 3. Due to a lack of engagement with the experimental task, those participants with zero variance in their responses (i.e., pressing the same button, for all nine of the geometric shapes tested in Experiment 3) were excluded from the study sample analysis, together with an outlier exclusion in the RT data (i.e., answers consisting of a single button being pressed, or RTs that were too slow/fast, e.g., RT*_z_*_-score _> ±3; Juravle & Spence, 2015; Pukelsheim, 1994). The remaining sample (*N *= 154, 17 males, one nonbinary) had a mean age of 24 years old (*SD *= 8 years, age range 20–50 years).

The apparatus and materials were as in Experiment 1. The study was conducted online on the Testable platform (www.testable.org; Rezlescu et al., 2020) between 1 September 2023 and 7 October 2023. The participants were informed that the experiment was designed to investigate any links that exist between geometric shapes and the perception of temperature and were asked to give their subjective appreciation of ice. They gave their consent to take part in the study. The participants received nine trials, each with a single 3D rotating geometric shape, and six conceptual attribute buttons, placed below the shape: solid, natural, bright, strong, crystalline, and beautiful. On each trial, the participants were instructed to choose the attribute that they thought was most representative for the currently displayed geometric shape, by clicking on one of the six available buttons. By forcing participants to choose a single attribute from the six presented concepts for their response, we assured the representativeness of their choice. They were informed that there were no correct or incorrect answers; we required their subjective opinion on the shape–conceptual attribute match. Once their choice had been made, the experiment continued on to the next trial. The order of the trials was randomized across participants.

Following the experimental trials, the participants were asked to fill in the various psychological scales, as used in Experiments 1 and 2: The scale assessing extroversion from the International Personality Item Pool, the total *extraversion* score had excellent internal consistency (Cronbach's α = .91; 95% CI [0.88, 0.93]); the *introversion* scale with a good Cronbach's α = .83 (95% CI [0.78, 0.87]); and the IFIS fitness score with good Cronbach's α = .84 (95% CI [0.79, 0.87]). The experiment took an average of 7 min to complete.

For each of the geometric shapes, frequency tables of the six conceptual attributes were calculated and then the Pearson χ^2^ test was used to compare the observed frequencies in the sample to those attributes' frequencies predicted by chance. For the analysis, we considered equal predicted frequencies for each of the conceptual attributes in each of the nine geometric shapes tested in Experiment 3 (i.e., each conceptual attribute with a predicted frequency of 16.67%).

### Results

**Figure 6.**
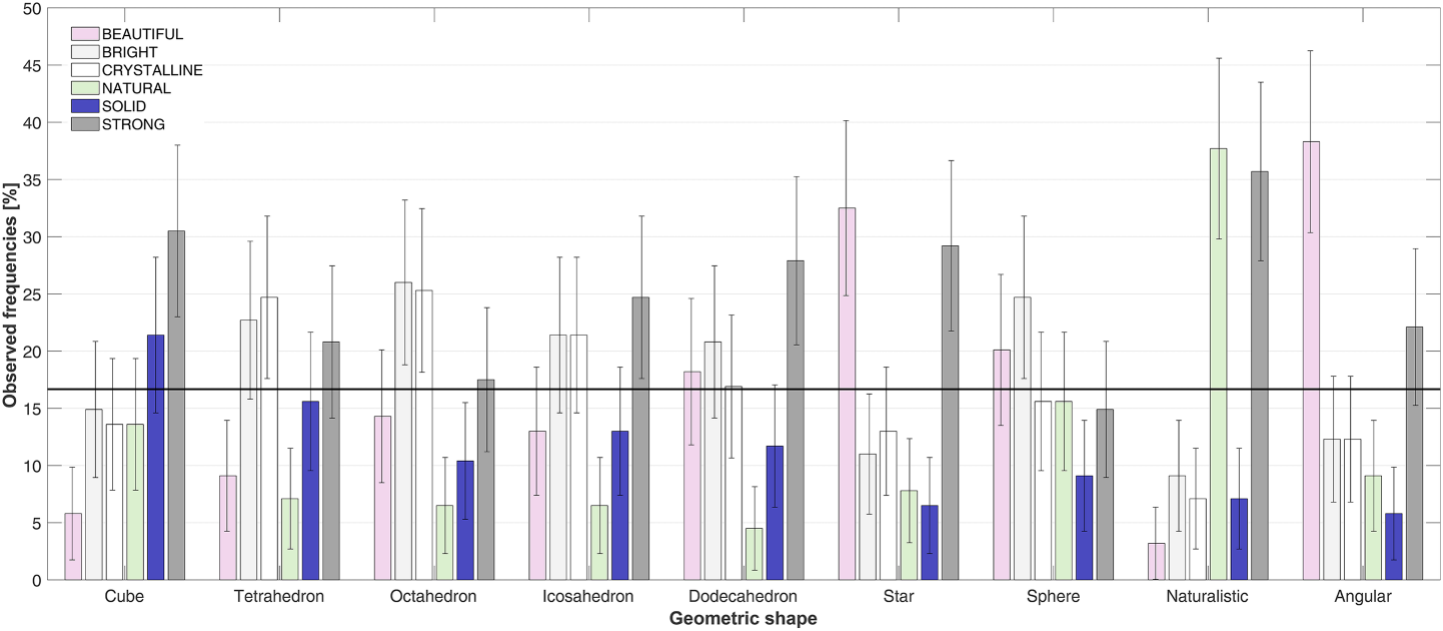
Means ± 95% CI (presented as percentages) for the appreciation of each of the six conceptual attributes assessed to represent each of the nine geometric shapes in Experiment 3. The participants were asked to pair one of the six attributes to each geometric shape presented. The thick horizontal line indicates chance-level responding (i.e., 16.67%) (refer to the online version of this graph for color version).

Figure 6 summarizes the data concerning the conceptual attributes (means ± 95% CI) in Experiment 3, for each of the nine geometric shapes. The shapes are ordered on the *x*-axis from the five Platonic solids (cube, tetrahedron, octahedron, icosahedron, and dodecahedron), followed by the regular star polyhedron, the sphere, with the naturalistic irregular shape and the angular irregular shape closing the line on the right of Figure 6. Visual inspection of the figure indicates that some of the assessed conceptual attributes failed to surpass chance (depicted by the thick black line, at 16.67%). The majority of the geometric shapes exhibited two conceptual attributes chosen at above chance levels, e.g., the cube, χ^2^(5) = 32.62; *p *< .001; the star polyhedron, χ^2^(5) = 58.65; *p *< .001; the sphere, χ^2^(5) = 12.83; *p *= .025; the naturalistic shape, χ^2^(5) = 112.96; *p *< .001; and the angular irregular shape, χ^2^(5) = 65.84; *p *< .001. Furthermore, a series of geometric shapes highlight three conceptual attributes chosen at above chance level, including the tetrahedron, χ^2^(5) = 24.68; *p *< .001; the octahedron, χ^2^(4) = 28.73; *p *< .001; the icosahedron, χ^2^(5) = 22.18; *p *< .001; and the dodecahedron, χ^2^(5) = 29.35; *p *< .001.

It is visually evident in Figure 6 that the only geometric shape surpassing chance level for the solid conceptual attribute is the cube. Furthermore, as expected, the Platonic solids are consistently conceptually appreciated as strong, given that all ratings of the Platonic solids are localized above the line representing chance-level performance. From among the Platonic solids, the tetrahedron, the octahedron, the icosahedron, and the dodecahedron are significantly considered as being bright, as is the sphere. The crystalline concept has been attributed to the octahedron and the icosahedron, whereas, unsurprisingly, the naturalistic stone-like geometric shape was the only shape with the natural appreciation rising above the chance-level line. Importantly, even though the beautiful conceptual appreciation was given above chance to several of the geometric shapes, including the rounded ones, such as the dodecahedron and the sphere, but also the star polyhedron and the angular irregular shape, the results of Experiment 3 indicate that the sphere was not appreciated as the most beautiful shape. Rather, it is the angular irregular shape and the star regular polyhedron that were rated as being most beautiful.

### Discussion

The results of Experiment 3 demonstrate that the Platonic solids are characterized conceptually as being strong, solid, bright, and crystalline. On the other hand, the results also highlight that for those cases where rounded objects are made of ice, their dominion in terms of beauty and liking appears to falter: The geometric shapes associated with the beautiful concept in Experiment 3 were the star polyhedron and the irregular angular shape, with both shapes resembling the typical formation of ice-shaped snowflakes. Both simple and complex 2D renderings of snowflakes have previously been associated with enhanced esthetic evaluations (Adkins & Norman, 2016; see also, Phillips et al., 2010). In the General Discussion, several explanations for these findings are considered and a few suggestions in terms of the potential applications of these findings are discussed.

## General Discussion

The present study investigated sensorial and conceptual attributes of ice-textured visually presented geometric shapes, by opposing the most liked geometric shape, the sphere, to other geometric shapes, namely the Platonic solids, long famous for their beauty. Presenting participants with shapes acknowledged as beautiful in a naturalistic state (i.e., made of ice) allowed us to describe ice both sensorially and in terms of its conceptual (connotative) associations. These results indicate comparable liking and hardness ratings for the sphere and the Platonic solids. The regular star polyhedron and the irregular star-like control shape were conceptually classified as beautiful and received the highest liking ratings in this study. As expected, all of the Platonic solids were considered strong, with the cube being classified as solid.

Symmetry-based studies have established the spherical shape together with the related rounded contours as typically being rated as beautiful and highly liked (Bar & Neta, 2006; Ghoshal et al., 2015; Silvia & Barona, 2009). By visually attaching the attribute of naturalness to the sphere, in the context of a rather icy presentation, these results demonstrate that liking, and the appreciation of beauty, is, to a certain degree at least, context-dependent. That is, when considering an ice-shaped sphere, we are faced with a trade-off between two incompatible features, as these are demonstrated in crossmodal research: We appreciate the rounded contour, but likely dislike the implied temperature of the ice finish. In a similar take on context in shape perception, it has recently been suggested that the preference for rounded/curved shapes is actually determined by presentation time, stimulus, and task type, as well as expertise with a certain stimulus (Chuquichambi et al., 2022), but also by taste expectations and the emotional valence of the visual 2D stimuli (Motoki & Velasco, 2021). Moreover, people would appear to differ significantly on important artistic sensitivity criteria, including symmetry, when appreciating beauty (Corradi et al., 2020; see also Corradi et al., 2019; Cotter et al., 2017).

Even though temperature was only suggested visually in the current study, through the visually derived ice finish of all geometric shapes used, our results indicate that the perceived/implied low ice temperature of the depicted objects impacted both participants' sensorial and conceptual consideration. The temperature of ice is an easily established visual characteristic, the certainty of which could not be changed, as is the case of other surface materials, that could feel cold/warm, but which need tactile input to ascertain their actual temperature. To include familiarity and naturalness in our discussion, a somewhat natural correspondence between lower temperatures and angularity could be implied, just by taking into consideration that water freezing transforms into regular angular crystalline structures. Nevertheless, note that our findings indicate that temperature was rightfully transmitted through the visual presentation of our stimuli: All of the geometric stimuli presented in Experiment 1 were rated as being colder in terms of estimated temperature (i.e., all average ratings fall below the middle of the temperature VAS). Relatedly, the shapes were consistently rated as wet and, aside from the naturalistic rock-like shape, all the other shapes were rated as significantly slippery. Taken together, these results of combined low temperature, enhanced wetness, and slipperiness highlight the successful visual rendition of ice in our study.

Considering the ice-texture finish of our stimuli, the results of Experiment 1 indicate that context may be implicated in shape perception (Motoki & Velasco, 2021). Presenting a sphere in a neutral gray hue makes people rate it as soft and warm, softer and warmer even than other symmetrical angular shapes having the same neutral gray hue (Juravle et al., 2022; see also Woods et al., 2013, for manipulations of expectation in color-shape correspondences). However, when adding a naturalistic context from the visually derived ice texture, as this was manipulated in the present study, the results highlight an equivalence in appreciating temperature, hardness, texture, and wetness. All these sensory qualities appear to be driven first and foremost by people's *knowledge* of the material properties of ice (see, e.g., Marschallek & Jacobsen, 2022, for the appreciation of various material qualities; and Spence, 2020a, for a review). Such findings suggest that (the perception of) shape may not be independent of the consideration of material qualities. Relatedly, context can also give way to emotional valence in the appreciation of shape. For example, a cold stimulus such as ice may be perceived as pleasant or unpleasant depending on the environmental context and the location on the body where it is experienced. Take the pleasant case of a glass of water with ice cubes, sipped during a hot summer day, highlighting our enjoyment of cold drinks (Eccles et al., 2013). On the other hand, iced water likely soon loses its appeal when served in a wintery environment.

Furthermore, it has been demonstrated that familiarity with an object and its attributes impacts its sensorial appreciation (Chuquichambi et al., 2021), as well as how people relate and interact with its various parts, e.g., we will fixate the top part of novel objects we come into contact with, but we will likely grasp them according to their center of mass (Juravle et al., 2015). In a similar vein, it appears that people are more likely to associate rounded shapes with the self, whereas angular ones are attributed to strangers (i.e., what has been termed the self-prioritization effect; Manippa & Tommasi, 2023; Vicovaro et al., 2022). Moreover, while the results reported here are reflective of people's subjective visual appreciation of ice-textured objects, future studies are needed in order to investigate the *objective feel* of ice and its impact in the assessment of various sensorial qualities, such as these were investigated in the present study (i.e., the appreciated temperature of a geometric shape). For example, people's liking of 3D objects differs somewhat, as a function of whether these shapes are presented visually or haptically (Etzi et al., 2012). However, their perceived temperature is still to be investigated (even though other temperature-based correspondences have received considerable attention in the literature; see, e.g., Spence, 2020b, for color-temperature correspondences). Furthermore, take, for instance, structural integrity characteristics such as compressibility and/or resistance to deformation (see, e.g., Tiest & Kappers, 2006), which may predict object intrinsic qualities like *solid* and/or *strong*, as they were attributed to the cube and naturalistic stone in Experiment 3. On the other hand, the concept of *bright* could be better predicted by surface qualities such as reflection or brilliance, which may reflect material qualities such as gloss (Chadwick & Kentridge, 2015), translucency, and/or transparency (Anderson, 2011), as these were attributed to the octahedron and the sphere in Experiment 3. Here, a geometric shape's complexity should also be considered, when ascertaining higher-order conceptual qualities of a given object. For instance, it has been shown that simple 2D geometric shapes are perceived to be glossier, as compared to more complex shapes (van Assen et al., 2016; see also Beck, 1964, 1965; Kaplan, 1969). Furthermore, it has been suggested that some complexity-driven properties of objects may weigh more in esthetic evaluations than others: Aside from objects' symmetry (Rosen, 2008), researchers have pointed the spatial complexity of objects (Papadimitriou, 2020a; 2020b), the geometry of angles (Bertol, 2016), and spatial entropy (Papadimitriou, 2022) and topology (Papadimitriou, 2023). Relatedly, because of their association with the sun, radiating qualities like radial symmetry or sphericity would also be expected to predict brilliance or warmth. In a similar vein, consideration needs to be given to the specific type of interaction with objects of interest. Note that the effect of temperature can be tracked even in the absence of a thermal sensory stimulus, e.g., consider here only how the human pupils have been reported to contract in response to the mere imagination of a light body, such as the sun (Laeng & Sulutvedt, 2014).

With respect to the individual differences investigated in the present study, our results indicate that personality traits interact with the appreciation of geometric ice structures, such that extroverts appreciate ice as bright and crystalline. A meta-analysis on Big Five extroversion and alcohol use found that higher extroversion was associated with a higher probability of drinking alcohol, higher alcohol use per occasion of drinking, and higher alcohol use over a specific time period (Kotov et al., 2010). Even though many distilled alcoholic beverages/spirits tend to be served with ice (i.e., on the rocks), an association between iced alcohol drinks and extroversion is still to be demonstrated. Furthermore, those people who consider themselves to be fit tend to also consider ice as beautiful and natural, and somehow mirroring the enhanced hardness of the cube in the strength component of physical fitness, our results indicate fitter (by self-report) people's superior preference for the ice cubes, as well as their reporting of an enhanced hardness of the sphere. It appears that the drive for thinness, a demonstrated indicator for an eating disorder tendency, is positively correlated with the degree of the classical crossmodal correspondence between sweet taste and rounded geometric contours (Hamamoto et al., 2020). Moreover, research highlights that people with body dysmorphic disorder (BDD) associate negative words to their selves (Rief et al., 2006; Veale, 2004). Considering that athletes are less likely to develop BDD and, hence, have a better body image (Ghasemi et al., 2010), and the typical pairing of negative emotions and angular shapes versus the positive emotions and round shapes, our results of enhanced hardness for the cube and the sphere in self-assessed fitter people could be taken to indicate the perceived sensorial hardness of objects as a positive quality. That is, if we were to consider the universal pairing of round and soft, for example, (cube)-angular and hard could be taken as the angular counterpart.

Taken together, the present study highlights the importance of context and semantic meaning in crossmodal shape perception. Context is derived from naturalness of material cues (i.e., ice, as visually manipulated/rendered in the experiments presented here), as well as their familiarity, together with people's propensity to perceive specific features of a given object, as this is facilitated through intrinsic individual personality traits and self-reported fitness. Future studies need to further assess natural material textures, their interactions with the various human senses, and their importance and types of involvement in crossmodal shape perception.

## Supplemental Material

sj-pptx-1-ipe-10.1177_20416695241303004 - Supplemental material for Beauty is context-dependent: Naturalness, familiarity, and semantic meaning influence the appreciation of geometric shapes Supplemental material, sj-pptx-1-ipe-10.1177_20416695241303004 for Beauty is context-dependent: Naturalness, familiarity, and semantic meaning influence the appreciation of geometric shapes by Georgiana Juravle and Charles Spence in i-Perception

## Notes

1. Our conceptualizing of naturalness is closely linked to the esthetics of form/geometric shapes, *regular* rounded or rectangular, as opposed to the natural *irregular*, often curving forms, also known as and referred to as organic, or biophilic (see, e.g., examples in Archisoup, 2023, https://www.archisoup.com/shapes-in-architecture).

2. Note that there exist many natural shapes that are considered and/or accepted as being beautiful, even though they may not be geometric shapes, but *organic* configurations and/or forms. For the present purpose, the focus of the investigation is specifically on *geometric shape*(*s*).

## References

[bibr1-20416695241303004] Adkins O. C. Norman J. F. (2016). The visual aesthetics of snowflakes. Perception, 45(11), 1304–1319. 10.1177/2041669516661122 27457713

[bibr2-20416695241303004] Anderson B. L. (2011). Visual perception of materials and surfaces. Current Biology, 21(24), R978–R983. 10.1016/j.cub.2011.11.022 22192826

[bibr3-20416695241303004] Archisoup. (2023). Shapes in architecture. https://www.archisoup.com/shapes-in-architecture

[bibr4-20416695241303004] Bar M. Neta M. (2006). Humans prefer curved visual objects. Psychological Science, 17(8), 645–648. 10.1111/j.1467-9280.2006.01759 16913943

[bibr5-20416695241303004] Barbosa Escobar F. Velasco C. Byrne D. V. Wang Q. J. (2023a). Crossmodal associations between visual textures and temperature concepts. Quarterly Journal of Experimental Psychology, 76(4), 731–761. 10.1177/17470218221096452 35414309

[bibr6-20416695241303004] Barbosa Escobar F. Velasco C. Byrne D. V. Wang Q. J. (2023b). Assessing mechanisms behind crossmodal associations between visual textures and temperature concepts. Journal of Experimental Psychology: Human Perception and Performance, 49(6), 923–947. 10.1037/xhp0001131 37276128

[bibr7-20416695241303004] Barbosa Escobar F. Velasco C. Byrne D. V. Wang Q. J. (2023c). The influence of emotional cues and anthropomorphism on product temperature expectations. PsyArXiv. 10.31234/osf.io/v6p2e

[bibr8-20416695241303004] Beck J. (1964). The effect of gloss on perceived lightness. The American Journal of Psychology, 77(1), 54–63. 10.2307/1419271 14130364

[bibr9-20416695241303004] Beck J. (1965). Apparent spatial position and the perception of lightness. Journal of Experimental Psychology, 69(2), 170–179. 10.1037/h0021571 14279774

[bibr10-20416695241303004] Bertol D. (2016). The parametric making of geometry: The Platonic solids. International Journal of Rapid Manufacturing, 6(1), 33–52. 10.1504/IJRAPIDM.2016.078743

[bibr11-20416695241303004] Chadwick A. C. Kentridge R. W. (2015). The perception of gloss: A review. Vision Research, 109(Part B), 221–235. 10.1016/j.visres.2014.10.026 25448119

[bibr12-20416695241303004] Chen N. Watanabe K. Wada M. (2021). People with high autistic traits show fewer consensual crossmodal correspondences between visual features and tastes. Frontiers in Psychology, 12, 714277. 10.3389/fpsyg.2021.71427 34566793 PMC8457010

[bibr13-20416695241303004] Chuquichambi E. G. Palumbo L. Rey C. Munar E. (2021). Shape familiarity modulates preference for curvature in drawings of common-use objects. PeerJ, 7, e11772. 10.7717/peerj.11772 PMC8269663 34268016

[bibr14-20416695241303004] Chuquichambi E. G. Vartanian O. Skov M. Corradi G. B. Nadal M. Silvia P. J. Munar E. (2022). How universal is preference for visual curvature? A systematic review and meta-analysis. Annals of the New York Academy of Sciences, 1518(1), 151–165. 10.1111/nyas.14919 36285721 PMC10091794

[bibr15-20416695241303004] Cohen J. (1988). Statistical power for the social sciences. Laurence Erlbaum and Associates.

[bibr16-20416695241303004] Corradi G. Belman M. Currò T. Chuquichambi E. G. Rey C. Nadal M. (2019). Aesthetic sensitivity to curvature in real objects and abstract designs. Acta Psychologica, 197, 124–130. 10.1016/j.actpsy.2019.05.012 31146089

[bibr17-20416695241303004] Corradi G. Chuquichambi E. G. Barrada J. R. Clemente A. Nadal M. (2020). A new conception of visual aesthetic sensitivity. British Journal of Psychology, 111(4), 630–658. 10.1111/bjop.12427 31587262

[bibr18-20416695241303004] Cotter K. N. Silvia P. J. Bertamini M. Palumbo L. Vartanian O. (2017). Curve appeal: Exploring individual differences in preference for curved versus angular objects. i-Perception, 8(2), 1–17. 10.1177/2041669517693023 PMC5405906 28491269

[bibr19-20416695241303004] Eccles R. Du-Plessis L. Dommels Y. Wilkinson J. E. (2013). Cold pleasure. Why we like ice drinks, ice-lollies and ice cream. Appetite, 71, 357–360. 10.1016/j.appet.2013.09.011 24060271

[bibr20-20416695241303004] Elliot A. J. Maier M. A. (2012). Color-in-context theory. Advances in Experimental Social Psychology, 45, 61–125. 10.1016/B978-0-12-394286-9.00002-0

[bibr21-20416695241303004] Erdfelder E. Faul F. Buchner A. Lang A. G. (2009). Statistical power analyses using G*Power 3.1: Tests for correlation and regression analyses. Behavior Research Methods, 41(4), 1149–1160. 10.3758/BRM.41.4.1149 19897823

[bibr22-20416695241303004] Etzi R. Spence C. Gallace A. (2012). Aesthetic preferences for tridimensional shapes: A comparison between vision and touch. Seeing & Perceiving, 25(Supple.), 131. 10.1163/187847612X647630

[bibr23-20416695241303004] Faul F. Erdfelder E. Lang A. G. Buchner A. (2007). G*Power 3: A flexible statistical power analysis program for the social, behavioral, and biomedical sciences. Behavior Research Methods, 39(2), 175–191. 10.3758/BF03193146 17695343

[bibr24-20416695241303004] Fujisaki W. (2020). Multisensory Shitsukan perception. Acoustical Science and Technology, 41(1), 189–195. 10.1250/ast.41.189

[bibr25-20416695241303004] Gallace A. Spence C. (2014). In touch with the future: The sense of touch from cognitive neuroscience to virtual reality. Oxford University Press.

[bibr26-20416695241303004] Ghasemi A. Ardakani Z. P. Momeni M. Falahati M. Azimzade E. (2010). The investigation of the relationship between body dysmorphic disorder and psychological problems and comprise it among female athletes and non athletes students. Procedia-Social and Behavioral Sciences, 5, 1799–1803. 10.1016/j.sbspro.2010.07.367

[bibr27-20416695241303004] Ghoshal T. Boatwright P. Malika M. (2015). Curvature from all angles: An integrative review and implications for product design. In Batra R. Seifert C. M. Brei D. E. (Eds.), The psychology of design: Creating consumer appeal (pp. 91–106). Routledge.

[bibr28-20416695241303004] Goldberg L. R. Johnson J. A. Eber H. W. Hogan R. Ashton M. C. Cloninger C. R. Gough H. G. (2006). The international personality item pool and the future of public-domain personality measures. Journal of Research in Personality, 40(1), 84–96. 10.1016/j.jrp.2005.08.007

[bibr29-20416695241303004] Gombrich E. H. (1960). On physiognomic perception. Daedalus, 89(1), 228–242.

[bibr30-20416695241303004] Gombrich E. H. (1972). The mask and the face: The perception of physiognomic likeness in life and art. In Gombrich E. H. Hochberg J. Black M. (Eds.), Art, perception, and reality (pp. 1–45). Johns Hopkins University Press.

[bibr31-20416695241303004] Guest S. Catmur C. Lloyd D. Spence C. (2002). Audiotactile interactions in roughness perception. Experimental Brain Research, 146, 161–171. 10.1007/s00221-002-1164-z 12195518

[bibr32-20416695241303004] Hamamoto Y. Motoki K. Sugiura M. (2020). Assessing the relationship between drive for thinness and taste-shape correspondences. Multisensory Research, 34(1), 69–92. 10.1163/22134808-bja10030 33706274

[bibr33-20416695241303004] Holm S. (1979). A simple sequential rejective multiple test procedure. Scandinavian Journal of Statistics, 6, 65–70.

[bibr34-20416695241303004] Huylebrouck D. (2023). A new family of solids: The infinite Kepler-Poinsot polyhedra. Qeios, 6(10). https://doi.org/10.32388/gek0y9

[bibr35-20416695241303004] Hyde R. J. Witherly S. A. (1993). Dynamic contrast: A sensory contribution to palatability. Appetite, 21(1), 1–16. 10.1006/appe.1993.1032 8239631

[bibr36-20416695241303004] Iliescu D. Popa M. Dimache R. (2015). Adaptarea românească a Setului International de Itemi de Personalitate: IPIP-Ro [The Romanian adaptation of the International Personality Item Pool: IPIP-Ro]. Psihologia Resurselor Umane, 13(1), 83–112.

[bibr37-20416695241303004] JASP Team. (2022). JASP (Version 0.16.1) [Computer software]. JASP – Free and User-Friendly Statistical Software.

[bibr38-20416695241303004] Juravle G. Duca R. Feghiuș C. Spence C. (2024). Hot and round: How temperature and shape impact the multisensory appreciation of cornmeal. International Journal of Gastronomy and Food Science, 35, 100893. 10.1016/j.ijgfs.2024.100893

[bibr39-20416695241303004] Juravle G. Olari E. L. Spence C. (2022). A taste for beauty: On the expected taste, hardness, texture, and temperature of geometric shapes. i-Perception, 13(5), 20416695221120948. 10.1177/20416695221120948 36157518 PMC9490474

[bibr40-20416695241303004] Juravle G. Spence C. (2015). Speed of reaction to sensory stimulation is enhanced during movement. Acta Psychologica, 161, 154–161. 10.1016/j.actpsy.2015.09.002 26398485

[bibr41-20416695241303004] Juravle G. Velasco C. Salgado-Montejo A. Spence C. (2015). The hand grasps the center, while the eyes saccade to the top of novel objects. Frontiers in Psychology, 6, 1–9. 10.3389/fpsyg.2015.00633 26052291 PMC4441126

[bibr42-20416695241303004] Kahrimanovic M. Bergmann Tiest W. M. Kappers A. M. L. (2010). Seeing and feeling volumes: The influence of shape on volume perception. Acta Psychologica, 134(3), 385–390. 10.1016/j.actpsy.2010.03.011 20421094

[bibr43-20416695241303004] Kaplan G. A. (1969). Kinetic disruption of optical texture: The perception of depth at an edge. Perception & Psychophysics, 6(4), 193–198. 10.3758/BF03207015

[bibr44-20416695241303004] Komatsu H. Goda N. (2018). Neural mechanisms of material perception: Quest on Shitsukan. Neuroscience, 392, 329–347. 10.1016/j.neuroscience.2018.09.001 30213767

[bibr45-20416695241303004] Kotov R. Gamez W. Schmidt F. Watson D. (2010). Linking ‘big’ personality traits to anxiety, depressive, and substance use disorders: A meta-analysis. Psychological Bulletin, 136(5), 768–821. 10.1037/a0020327 20804236

[bibr46-20416695241303004] Laeng B. Sulutvedt U. (2014). The eye pupil adjusts to imaginary light. Psychological Science, 25(1), 188–197. 10.1177/0956797613503556 24285432

[bibr47-20416695241303004] Liu C. H. Kennedy J. M. (1993). Symbolic forms and cognition. Psyke and Logos, 14, 441–456.

[bibr48-20416695241303004] Liu C. H. Kennedy J. M. (1997). Form symbolism, analogy, and metaphor. Psychonomic Bulletin & Review, 4, 546–551. 10.3758/BF03214347

[bibr49-20416695241303004] Lloyd D. R. (2010). Symmetry and beauty in Plato. Symmetry, 2(2), 455–465. 10.3390/sym2020455

[bibr50-20416695241303004] MacGregor G. (2000). Making sense of the past in the present: A sensory analysis of carved stone balls. World Archaeology, 31(2), 258–271. 10.1080/00438243.1999.9980445

[bibr51-20416695241303004] Manippa V. Tommasi L. (2023). The shape of you: Do individuals associate particular geometric shapes with identity? Current Psychology, 42(12), 1–11. 10.1007/s12144-021-02297-z 33519148

[bibr52-20416695241303004] Marks L. E. (2014). The unity of the senses: Interrelations among the modalities. Academic Press.

[bibr53-20416695241303004] Marschallek B. E. Jacobsen T. (2022). Materials aesthetics: A replication and extension study of the conceptual structure. PLoS One, 17(11), e0277082. 10.1371/journal.pone.0277082 PMC9629638 36322590

[bibr54-20416695241303004] McCrae R. R. John O. P. (1992). An introduction to the five-factor model and its applications. Journal of Personality, 60(2), 175–215. 10.1111/j.1467-6494.1992.tb00970.x 1635039

[bibr55-20416695241303004] Melo I. (2022). Aesthetic criteria in fundamental physics—The viewpoint of Plato. Philosophies, 7(5), 96. 10.3390/philosophies7050096

[bibr56-20416695241303004] Motoki K. Velasco C. (2021). Taste-shape correspondences in context. Food Quality and Preference, 88, 104082. 10.1016/j.foodqual.2020.104082

[bibr57-20416695241303004] Muller A. Barsky D. Sala-Ramos R. Sharon G. Titton S. Vergès J.-M. Grosman L. (2023). The limestone spheroids of ‘Ubeidiya: Intentional imposition of symmetric geometry by early hominins? Royal Society Open Science, 10(9), 230671. 10.1098/rsos.230671 37680494 PMC10480702

[bibr58-20416695241303004] Ortega F. B. Leskošek B. Blagus R. Gil-Cosano J. J. Mäestu J., Tomkinson G. R., Ruiz, J. R., Mäestu, E., Starc, G., Milanovic, I., Tammelin, T. H., Sorić, M., Scheuer, C., Carraro, A., Kaj, M., Csányi, T., Sardinha, L. B., Lenoir, M., Emeljanovas, A., … Jurak G. (2023). European fitness landscape for children and adolescents: Updated reference values, fitness maps and country rankings based on nearly 8 million test results from 34 countries gathered by the FitBack network. British Journal of Sports Medicine, 57(5), 299–310. 10.1136/bjsports-2022-106176 36623866 PMC9985767

[bibr59-20416695241303004] Ortega F. B. Ruiz J. R. España-Romero V. Vicente-Rodriguez G. Martínez-Gómez D., Manios Y., Béghin, L., Molnar, D., Widhalm, K., Moreno, L. A., Sjöström, M., Castillo M. J. , on behalf of the HELENA study group. (2011). The International Fitness Scale (IFIS): Usefulness of self-reported fitness in youth. International Journal of Epidemiology, 40(3), 701–711. 10.1093/ije/dyr039 21441238

[bibr60-20416695241303004] Osgood C. E. (1952). The nature and measurement of meaning. Psychological Bulletin, 49(3), 197–237. 10.1037/h0055737 14930159

[bibr61-20416695241303004] Osgood C. E. Suci G. J. Tannenbaum P. H. (1957). The measurement of meaning. University of Illinois Press.

[bibr62-20416695241303004] Papadimitriou F. (2020a). Exploring spatial complexity in 3d. In Spatial complexity (pp. 101–113). Springer. 10.1007/978-3-030-59671-2_7

[bibr63-20416695241303004] Papadimitriou F. (2020b). Spatial complexity, visual complexity and aesthetics. In Spatial complexity (pp. 243–261). Springer. 10.1007/978-3-030-59671-2_16

[bibr64-20416695241303004] Papadimitriou F. (2022). Visual perception of spatial entropy and landscape analysis. In Spatial entropy and landscape analysis. RaumFragen: Stadt – Region – Landschaft (pp. 87–102). Springer VS. 10.1007/978-3-658-35596-8_6

[bibr65-20416695241303004] Papadimitriou F. (2023). Geo-topology and visual impact. In Geo-topology. GeoJournal library (Vol. 133, pp. 139–150). Springer. 10.1007/978-3-031-48185-7_11

[bibr66-20416695241303004] Phillips F. Norman J. F. Beers A. M. (2010). Fechner’s aesthetics revisited. Seeing and Perceiving, 23(3), 263–271. 10.1163/187847510X516412 20819476

[bibr67-20416695241303004] Pukelsheim F. (1994). The three sigma rule. The American Statistician, 48(2), 88–91. 10.1080/00031305.1994.10476030

[bibr68-20416695241303004] Rezlescu C. Danaila I. Miron A. Amariei C. (2020). More time for science: Using testable to create and share behavioral experiments faster, recruit better participants, and engage students in hands-on research. Progress in Brain Research, 253, 243–262. 10.1016/bs.pbr.2020.06.005 32771126

[bibr69-20416695241303004] Rief W. Buhlmann U. Wilhelm S. Borkenhagen A. D. A. Brähler E. (2006). The prevalence of body dysmorphic disorder: A population-based survey. Psychological Medicine, 36(6), 877–885. 10.1017/S0033291706007264 16515733

[bibr70-20416695241303004] Rosen J. (2008). Symmetry rules: How science and nature are founded on symmetry. Springer Science & Business Media.

[bibr71-20416695241303004] Silvia P. J. Barona C. M. (2009). Do people prefer curved objects? Angularity, expertise, and aesthetic preference. Empirical Studies of the Arts, 27(1), 25–42. 10.2190/em.27.1.b

[bibr72-20416695241303004] Spence C. (2020a). Shitsukan—The multisensory perception of quality. Multisensory Research, 33(7), 737–775. 10.1163/22134808-bja10003 32143187

[bibr73-20416695241303004] Spence C. (2020b). Temperature-based crossmodal correspondences: Causes & consequences. Multisensory Research, 33, 645–682. 10.1163/22134808-20191494 31923885

[bibr74-20416695241303004] Spence C. (2022a). Exploring group differences in the crossmodal correspondences. Multisensory Research, 35(6), 495–536. 10.1163/22134808-bja10079 35985650

[bibr75-20416695241303004] Spence C. (2022b). What is the link between personality and food behavior? Current Research in Food Science, 5, 19–27. 10.1016/j.crfs.2021.12.001 34917953 PMC8666606

[bibr76-20416695241303004] Spence C. Zampini M. (2006). Auditory contributions to multisensory product perception. Acta Acustica United with Acustica, 92(6), 1009–1025.

[bibr77-20416695241303004] Tiest W. M. B. Kappers A. M. (2006). Analysis of haptic perception of materials by multidimensional scaling and physical measurements of roughness and compressibility. Acta Psychologica, 121(1), 1–20. 10.1016/j.actpsy.2005.04.005 16055070

[bibr78-20416695241303004] Tuovinen S. Tang X. Salmela-Aro K. (2020). Introversion and social engagement: Scale validation, their interaction, and positive association with self-esteem. Frontiers in Psychology, 11, 3241. 10.3389/fpsyg.2020.590748 PMC7734327 33329251

[bibr79-20416695241303004] Turoman N. Velasco C. Chen Y.-C. Huang P. C. Spence C. (2018). Symmetry and its role in the crossmodal correspondence between shape and taste. Attention, Perception, and Psychophysics, 80(3), 738–751. 10.3758/s13414-017-1463-x 29260503

[bibr80-20416695241303004] van Assen J. J. R. Wijntjes M. W. Pont S. C. (2016). Highlight shapes and perception of gloss for real and photographed objects. Journal of Vision, 16(6), 6–6. 10.1167/16.6.6 27271808

[bibr81-20416695241303004] Veale D. (2004). Advances in a cognitive behavioural model of body dysmorphic disorder. Body Image, 1(1), 113–125. 10.1016/S1740-1445(03)00009-3 18089145

[bibr82-20416695241303004] Vicovaro M. Dalmaso M. Bertamini M. (2022). Towards the boundaries of self-prioritization: Associating the self with asymmetric shapes disrupts the self-prioritization effect. Journal of Experimental Psychology: Human Perception and Performance, 48(9), 972–986. 10.1037/xhp0001036 35816564

[bibr83-20416695241303004] Wilczek F. (2015). A beautiful question: Finding nature’s deep design. Penguin Random House.

[bibr84-20416695241303004] Woods A. T. Spence C. Butcher N. Deroy O. (2013). Fast lemons and sour boulders: Testing crossmodal correspondences using an internet-based testing methodology. i-Perception, 4(6), 365–379. 10.1068/i0586 PMC3859554 24349696

